# Networking of differentially expressed genes in human cancer cells resistant to methotrexate

**DOI:** 10.1186/gm83

**Published:** 2009-09-04

**Authors:** Elisabet Selga, Carlota Oleaga, Sara Ramírez, M Cristina de Almagro, Véronique Noé, Carlos J Ciudad

**Affiliations:** 1 Department of Biochemistry and Molecular Biology, School of Pharmacy, University of Barcelona, Diagonal Avenue, E-08028 Barcelona, Spain

## Abstract

**Background:**

The need for an integrated view of data obtained from high-throughput technologies gave rise to network analyses. These are especially useful to rationalize how external perturbations propagate through the expression of genes. To address this issue in the case of drug resistance, we constructed biological association networks of genes differentially expressed in cell lines resistant to methotrexate (MTX).

**Methods:**

Seven cell lines representative of different types of cancer, including colon cancer (HT29 and Caco2), breast cancer (MCF-7 and MDA-MB-468), pancreatic cancer (MIA PaCa-2), erythroblastic leukemia (K562) and osteosarcoma (Saos-2), were used. The differential expression pattern between sensitive and MTX-resistant cells was determined by whole human genome microarrays and analyzed with the GeneSpring GX software package. Genes deregulated in common between the different cancer cell lines served to generate biological association networks using the Pathway Architect software.

**Results:**

Dikkopf homolog-1 (*DKK1*) is a highly interconnected node in the network generated with genes in common between the two colon cancer cell lines, and functional validations of this target using small interfering RNAs (siRNAs) showed a chemosensitization toward MTX. Members of the UDP-glucuronosyltransferase 1A (*UGT1A*) family formed a network of genes differentially expressed in the two breast cancer cell lines. siRNA treatment against *UGT1A *also showed an increase in MTX sensitivity. Eukaryotic translation elongation factor 1 alpha 1 (*EEF1A1*) was overexpressed among the pancreatic cancer, leukemia and osteosarcoma cell lines, and siRNA treatment against *EEF1A1 *produced a chemosensitization toward MTX.

**Conclusions:**

Biological association networks identified *DKK1*, *UGT1A*s and *EEF1A1 *as important gene nodes in MTX-resistance. Treatments using siRNA technology against these three genes showed chemosensitization toward MTX.

## Background

The large amount of information obtained with high-throughput technologies like expression microarrays needs to be processed in order to be comprehensible to molecular biologists. In this regard, many computational methods have been developed to facilitate expression data analysis. Gene clustering, gene ontology and pathway analyses are commonly used [1,2]. Pathways are manually generated diagrams that represent knowledge on molecular interactions and reactions [3] and they can be used to visualize the involvement of the differentially expressed genes in specific molecular, cellular or biological processes. However, the complexity of higher organisms cannot be explained solely as a collection of separate parts [4]; in organisms, pathways never exist in isolation, they are part of larger networks, which are more informative and real [5]. Gene networks are capable of describing a large number of interactions in a concise way, and provide a view of the physiological state of an organism at the mRNA level. Biochemical networks can be constructed at several levels and can represent different types of interactions. Literature mining allows the extraction of meaningful biological information from publications to generate networks [6]. Taking into account the progress in gene expression profiling, elucidating gene networks is an appropriate and timely step on the way to uncovering the complete biochemical networks of cells [5].

In this work, we use biological association networks (BANs) as a tool to define possible targets for gene therapy in combination with methotrexate (MTX). This approach could serve to minimize the development of MTX resistance acquired by cancer cells, which remains a primary cause of therapy failure in cancer treatment [7]. A role in MTX resistance was established for the three node genes selected, namely those encoding Dikkopf homolg 1 (*DKK1*), UDP-glucuronosyltransferases (UGTs; *UGT1A*s) and Eukaryotic translation elongation factor 1A1 (*EEF1A1*).

## Methods

### Cell lines

Cell lines representative of five types of human cancer were used: HT29 and Caco-2 for colon cancer, MCF-7 and MDA-MB-468 for breast cancer, MIA PaCa-2 for pancreatic cancer, K562 for erythroblastic leukemia, and Saos-2 for osteosarcoma. These cell lines are sensitive to MTX, with IC50s of 1.67 × 10^-8 ^M MTX for HT29, 4.87 × 10^-8 ^M MTX for MDA-MB-468 and 1.16 × 10^-8 ^M MTX for MIA PaCa-2 cells. IC50 values were calculated using GraphPad Prism 5 version 5.0a for Macintosh (GraphPad Software, San Diego, CA, USA). Resistant cells were obtained in the laboratory upon incubation with stepwise concentrations of MTX (Lederle) as previously described [8]. HT29, Caco-2 and K562 resistant cells were able to grow in 10^-5 ^M MTX; MIA PaCa-2, Saos-2, MCF-7 and MDA-MB-246 cells were resistant to 10^-6 ^M MTX.

### Cell culture

Human cell lines were routinely grown in Ham's F12 medium supplemented with 7% fetal bovine serum (both from Gibco/Invitrogen, Grand Island, NY, USA) at 37°C in a 5% CO_2 _humidified atmosphere. Resistant cells were routinely grown in selective DHFR medium lacking glycine, hypoxanthine and thymidine (-GHT medium; Gibco), the final products of dihydrofolate reductase (DHFR) activity. This medium was supplemented with 7% dialyzed fetal bovine serum (Gibco).

### Microarrays

Gene expression was analyzed by hybridization to the GeneChip^® ^Human Genome U133 PLUS 2.0 from Affymetrix, containing over 54,000 transcripts and variants. Total RNA for oligo arrays was prepared from triplicate samples of every sensitive and resistant cell line using the RNAeasy Mini kit (Qiagen, Germantown, Maryland, USA) following the recommendations of the manufacturer. Labeling, hybridization and detection were carried out following the manufacturer's specifications.

### Microarray data analyses

Gene expression analyses were performed using three samples of both sensitive and resistant cells for each of the seven cell lines studied. These analyses were carried out with the GeneSpring GX software v 7.3.1 (Agilent Technologies, Santa Clara, CA, USA), using the latest gene annotations available (March 2009). This software package allows multi-filter comparisons using data from different experiments to perform the normalization, generation of restriction (filtered) lists and functional classifications of the differentially expressed genes. Normalization was applied in two steps: 'per chip normalization', by which each measurement was divided by the 50th percentile of all measurements in its array; and 'per gene normalization', by which all the samples were normalized against the median of the control samples (sensitive cells). The expression of each gene was reported as the ratio of the value obtained for each condition relative to the control condition after normalization of the data. Then, data were filtered using the control strength, a control value calculated using the Cross-Gene Error model on replicates [9] and based on average base/proportional value. Measurements with higher control strength are relatively more precise than measurements with lower control strength. Genes that did not reach this value were discarded. Additional filtering was performed to determine differentially expressed genes. A first filter was performed by selecting the genes that displayed a *P*-value corrected by false discovery rate (Benjamini and Hochberg false discovery rate) of less than 0.05. The output of this analysis was then filtered by fold expression. Thus, lists of genes differentially expressed by at least twofold were generated for each of the seven resistant cell lines.

### Common genes between cell lines

The lists of genes differentially expressed by at least twofold with a *P*-value < 0.05 including multiple testing correction for each cell line were divided into two groups: overexpressed and underexpressed genes. Comparisons of lists of overexpressed genes were performed using Venn diagrams in GeneSpring GX. Lists of underexpressed genes were also compared using the same approach. All lists were compared in pairs and lists of genes in common between each pair were generated.

### Generation of biological association networks

BANs were constructed with the aid of Pathway Architect software v3.0 (Stratagene-Agilent). Briefly, this software package generates interaction networks starting with the genes in a given list (entities) taking into account the information present in a database of known molecular interactions. The lists correspond to the collection of differentially expressed genes under specific conditions. The database of molecular interactions is composed of more than 1.6 million interactions divided into different classes (binding, regulation, promoter binding, transport, metabolism, protein metabolism and expression). The interactions are extracted from the literature using a Natural Language Processing (NLP) tool run on Medline abstracts (NLP references), plus those obtained from external curated databases like BIND (Biomolecular Interaction Network Database) [10] and MINT (Molecular INTeraction) [11]. Interactions in the interaction database are scored according to five different categories: maximum, high, medium, low and minimal. Curated interactions (BIND and MINT sources) are given the maximum quality score, as are any interactions that have at least three NLP references. Pathway Architect gathers all that information to construct novel views as to how the entities in a list could be interacting with each other, even including entities not present in the original list (neighbors resulting from the expanded interaction). Customized analyses were performed to select relevant interaction networks with an associated high confidence index since such networks are likely to mirror biological significance. One-step expansion (using the expand network command) of the original set of entities with maximum score interaction were then analyzed by setting an advanced filter that included the categories of binding, expression, metabolism, promoter binding, protein modification and regulation. This procedure gives a final representation formed of a collection of nodes with different degrees of interrelationship. Some gene products from the original lists were not significantly connected with other members or neighbors and, therefore, were removed from the final view. Finally, members of the network were matched with expression levels.

### Transfection of small interfering RNAs against selected genes

HT29 cells (30,000) were plated in 1 ml of -GHT medium and transfection was performed 18 hours later. For each well, Lipofectamine™ 2000 (Invitrogen, Carlsbad, CA, USA) in 100 μl of serum free -GHT medium was mixed in Eppendorf tubes with 100 nM of small interfering RNA (siRNA) in 100 μl of serum free -GHT medium. The mixture was incubated at room temperature for 20 minutes before addition to the cells. MTX (2 × 10^-8 ^M) was added 48 hours after siRNA treatment and 3-(4,5-Dimethylthiazol-2-yl)-2,5-diphenyltetrazolium bromide (MTT) assays [12] were performed 3 days after MTX addition. Treatment of MDA-MB-468 and MIA PaCa-2 cells was performed following the same protocol but using Metafectene™ (Biontex, Martinsried, Germany). A non-related siRNA was used as negative control; it was transfected in parallel with the other siRNAs and used to normalize the results.

The siRNAs were designed using the software iRNAi v2.1. (The Netherlands Cancer Institute, Amsterdam, The Netherlands) Among the possible alternatives, sequences rich in A/T at the 3' end of the target were chosen. Then, BLAST resources in NCBI were used to assess the degree of specificity of the sequence recognition for these siRNAs. Only the siRNAs that reported the target gene as the only mRNA hit, or some family members in the case of siUGT1A, were selected. The sequences for the sense strand of all siRNAs used are available in Table 1.

**Table 1:** Sense strand sequences of the siRNAs used

Target gene	siRNA name	siRNA sequence (5'- 3')
*DKK1*	siDKK1	AGGTGCTGCACTGCCTATT
*UGT1A* family	siUGT1A	GTGCTGGGCAAGTTTACTT
*EEF1A1*	siEEF1A1	CGGTCTCAGAACTGTTTGT
*DHFR*	siDHFR	CCTCCACAAGGAGCTCATT
*Luciferase*	NR-siRNA	TAAGGCTATGAAGAGATAC

The sequences for the sense strand of the siRNAs used against the target genes are provided.

### Heat map generation

A global comparison of all cell lines was performed using GeneSpring GX v 7.3.1. The triplicate samples for each condition in each of the seven cell lines (42 samples) were imported into one single experiment. Normalization was performed in two steps: 'per chip normalization' (as described above) and 'per gene normalization', by which the samples were normalized against the median of all samples. Lists of genes displaying a false discovery rate-corrected *P*-value < 0.05 were generated for each cell line. As a filter, these values had to appear in at least two out of the seven cell lines. A hierarchical clustering method in GeneSpring was used to group genes on the basis of similar expression patterns over all samples. The distance matrix used was Pearson correlation, and average linkage was used as clustering algorithm. The same clustering method was used to group the cell lines on the basis of similar patterns of gene expression.

### Real-time RT-PCR

Gene mRNA levels were determined by real-time RT-PCR. Total RNA was extracted from cells using Ultraspec™ RNA reagent (Biotecx, Houston, TX, USA) following the recommendations of the manufacturer. For determining gene-node mRNA levels upon siRNA treatment, cells were treated as described above and total RNA was prepared 48 hours after transfection using the same reagent. In either case, complementary DNA was synthesized in a total volume of 20 μl by mixing 500 ng of total RNA and 125 ng of random hexamers (Roche, Mannheim, Germany) in the presence of 75 mM KCl, 3 mM MgCl_2_, 10 mM dithiothreitol, 20 units of RNasin (Promega, Madison, WI, USA), 0.5 mM dNTPs (AppliChem, Darmstadt, Germany), 200 units of M-MLV reverse transcriptase (Invitrogen) and 50 mM Tris-HCl buffer, pH 8.3. The reaction mixture was incubated at 37°C for 60 minutes. The cDNA product was used for subsequent real-time PCR amplification using an ABI Prism 7000 Sequence Detection System (Applied Biosystems, Foster City, CA, USA) with 25 ng of the cDNA mixture, the assays-on-demand from Applied Biosystems Hs00758822_s1 for DHFR and Hs00356991_m1 for Adenine Phosphoribosyltransferase (APRT) or the following primers: *DKK1*, 5'-AGTACTGCGCTAGTCCCACC-3' and 5'-CTGGAATACCCATCCAAGGTGC-3'; *UGT1A*, 5'-TAAGTGGCTACCCCAAAACG-3' and 5'-CTCCAGCTCCCTTAGTCTCC-3'; *EEF1A1*, 5'-CGTCATTGGACACGTAGATTCGGG-3' and 5'-GGAGCCCTTTCCCATCTCAGC-3'.

### Gene copy number determination

Genomic DNA from either sensitive or resistant cells was obtained with the Wizard™ Genomic DNA Purification Kit (Promega) following the manufacturer's recommendations. One hundred nanograms of DNA and the assays-on-demand Hs00758822_s1 for DHFR and Hs99999901_s1 for 18S were used for real-time PCR amplification.

### Preparation of total extracts for western blotting

Total extracts from cells, either sensitive or MTX-resistant, were used to assay DHFR protein levels. Cells were washed twice with ice-cold phosphate-buffered saline and scraped in 200 ml lysis buffer (50 mM Hepes, 500 mM NaCl, 1.5 M MgCl_2_, 1 mM EGTA, 10% glycerol (v/v), 1% Triton X-100 and protease inhibitor cocktail). Cells were incubated in ice for 1 hour with vortexing every 15 minutes and then centrifuged at 14,000 rpm at 4°C for 10 minutes. Five microliters of the extract were used to determine protein concentration by the Bradford assay (Bio-Rad, Munich, Germany). The extracts were frozen in liquid N_2 _and stored at -80°C. Fifty micrograms of both sensitive and resistant cell total extracts were resolved by 15% SDS-PAGE. Transference to PVDF membranes (Immobilon P, Millipore, Bedford, MA, USA) using a semidry electroblotter was followed by incubation with an antibody against DHFR (Davids Biotechnologie, Regensburg, Germany), and detection was accomplished using secondary horseradish peroxidase-conjugated antibody and enhanced chemiluminescence, as recommended by the manufacturer (Amersham/GE Healthcare, Buckinghamshire, UK). To normalize the results, blots were re-probed with an antibody against actin (Sigma, St. Louis, MO, USA).

### Transfections, co-transfections and luciferase assays

HT29 cells, either sensitive or MTX-resistant, were seeded into six-well plates the day before transfection at a density of 2 × 10^5 ^cells/well in Ham's F12 medium containing 7% fetal bovine serum. Transfection was performed using FUGENE™ HD (Roche). For each well, 6 μl of FUGENE™ HD in 100 μl of serum-free medium was incubated at room temperature for 5 minutes. The mixture was added to 1 μg of TOPFLASH (Millipore) and incubated at room temperature for 20 minutes before addition to the cells. In co-transfections, 1 μg of TOPFLASH was mixed together with 2 μg of pBATEM2-CDH before the addition of FUGENE™ HD in serum-free medium. The total amount of DNA was kept constant at 3 μg, adding empty vector when necessary. Luciferase activity was assayed 30 hours after transfection.

In all cases, cell extracts were prepared by lysing the cells with 200 μl of freshly diluted 1× Reporter Lysis Buffer (Promega). The lysate was centrifugated at 13,000 g for 2 minutes to pellet the cell debris and the supernatants were transferred to a fresh tube. A 15-μl aliquot of the extract was added to 15 μl of the luciferase assay substrate (Promega) and the luminiscence of the samples was read immediately on a Gloomax 20/20 luminometer (Promega); light production (relative light units) was measured for 10 s. Each transfection was performed in triplicate. Protein concentration was determined by the Bradford assay and used to normalize the results.

### Statistical analyses

Data are presented as mean ± standard error (SE). Statistical analyses were performed using the unpaired *t*-test option in GraphPad InStat version 3.1a for Macintosh (GraphPad Software). *P*-values < 0.05 were considered to be statistically significant.

## Results

### Genes deregulated in methotrexate-resistant cancer cell lines

In a previous study, we analyzed the differential gene expression between sensitive and MTX-resistant cells derived from the human colon cancer cell line HT29 [13]. In the present work we extend the study of gene expression profiles associated with MTX resistance by including another six MTX-resistant cell lines. Together, the studied cell lines represent colon cancer (CaCo2 and HT29), breast cancer (MCF-7 and MDA-MB-468), pancreatic cancer (MIA PaCa-2), erythroblastic leukemia (K562) and osteosarcoma (SaOs-2). Total RNA was extracted for the seven pairs of sensitive and MTX-resistant cell lines, and the expression profile of the 54,700 transcripts and variants included in the HG U133 PLUS 2.0 microarray from Affymetrix was compared between each pair using GeneSpring GX software v7.3.1. Upon normalization and statistical filtering of the data, lists of genes differentially expressed by at least twofold were built as described in Methods. These lists are presented as Additional data files 1 to 7. The data discussed in this report have been deposited in the Gene Expression Omnibus (GEO) [14] and are accessible through GEO series accession number [GSE16648].

### Hierarchical clustering of genes and cell lines

**Figure 1:**
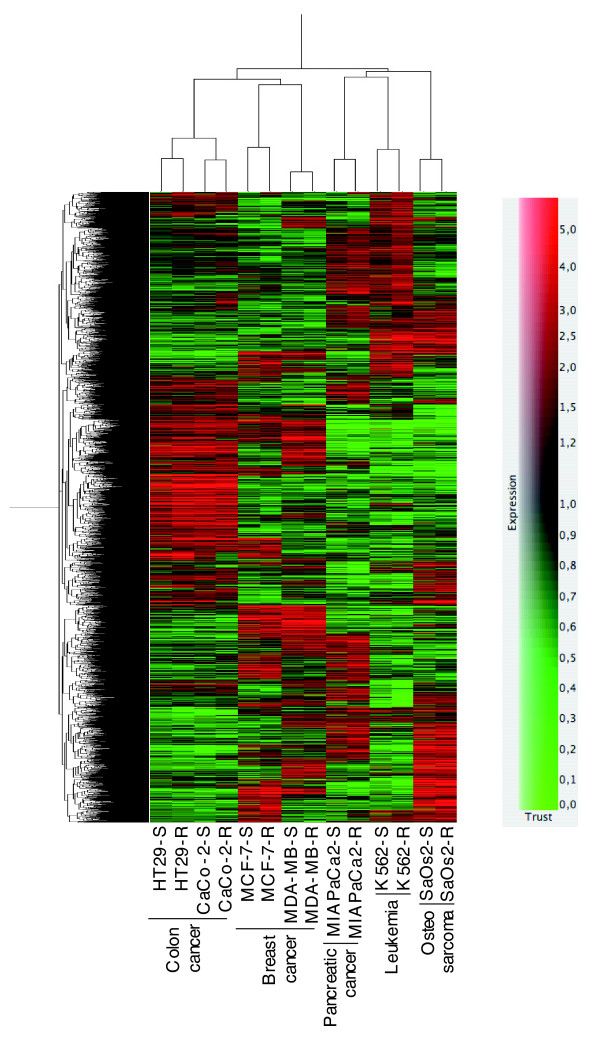
Heat map of differentially expressed genes. Lists of differentially expressed genes with a *t*-test *P*-value < 0.05 including multiple testing correction were generated for each cell line. A hierarchical clustering method in GeneSpring GX v 7.3.1 was used to construct both the gene tree and the sample tree, as described in Methods. Data are shown in a matrix format: each row represents a single gene, and each column represents a cell line. Red indicates overexpressed genes (expression levels over the median) and green indicates underexpressed genes (expression levels under the median; see legend). The pattern and length of the branches in the dendrograms reflect the relatedness of the samples or the genes.

We compared the gene expression patterns of all the studied cell lines together. Lists of genes displaying a false discovery rate-corrected *P*-value < 0.05 were generated for each cell line. Then, hierarchical clustering in GeneSpring GX was used to construct a heat map displaying both a gene tree and a sample tree (Figure 1), as described in Methods. Two facts could be extracted from this representation. First, there is a high correlation between cell lines sharing the same tissue origin. The two colon cancer cell lines studied (HT29 and Caco-2) are more highly correlated in gene expression with each other than with all the other cell lines. The breast cancer cell lines studied (MCF-7 and MDA-MB-468) also showed similar gene expression, although the degree of correlation is slightly lower than that for the colon cancer cell lines. The other three cell lines studied (MIA PaCa-2, K562 and Saos-2) displayed different gene expression from the colon or the breast cancer cell lines, and thus cluster apart from them. Second, gene expression of the resistant cells is more closely correlated with that of their sensitive cell counterparts than with any other sample or cell line.

### Dihydrofolate reductase status in all cell lines studied

As DHFR is the target for MTX, and was upregulated in MTX-resistant cells, we validated its overexpression in all cell lines studied. Real-time RT-PCR was used to quantify the mRNA levels, and DHFR protein levels were determined by western blotting in both sensitive and MTX-resistant cell lines (Table 2). Copy number determination revealed *dhfr *amplification only in HT29, Caco-2, MCF-7 and MIA PACA-2 resistant cells (Table 2).

**Table 2:** Validation of *dhfr *overexpression and copy number determination in the different cell lines

	Expression			
			
Cell line	Microarray	RT-PCR validation	Copy number	Protein
HT29	7.1	10.8 ± 0.7	16.1 ± 1.4	++
Caco-2	46.7	49.7 ± 1.1	83.4 ± 8.1	ND
MCF-7	31.1	33.2 ± 0.7	58.1 ± 0.8	+++++
MDA-MB-468	1.8	3.4 ± 0.1	0.9 ± 0.1	ND
MIA PaCa-2	9.5	8.2 ± 1.1	32.2 ± 2.2	+++
K562	9.4	9.8 ± 0.2	1.9 ± 0.1	++++
Saos-2	4.1	4.1 ± 1.1	0.6 ± 0.1	+

The overexpression of DHFR was validated at the mRNA and protein levels by real-time RT-PCR and western blotting using a specific antibody, respectively. DHFR expression levels are presented both as the values found in the microarrays and as validated by RT-PCR. *dhfr *copy number was determined by real-time PCR. Values are the mean (in fold change relative to the sensitive cells) of three independent experiments ± SE. Protein levels were qualitatively assessed (+ to +++++) from the western blot images. ND, non-determined.

### Identification of genes differentially expressed in common among different cell lines resistant to MTX

Lists of genes differentially expressed by at least twofold between sensitive and resistant cell lines were generated for each cell line. Each list was split in two, one group including the overexpressed genes and the other including the underexpressed genes. Then, Venn diagrams were used to compare the lists of overexpressed and underexpressed genes between HT29 and Caco-2 cell lines, between MCF-7 and MDA-MB-468 cell lines, and among MIA PaCa-2, K562 and Saos-2 cell lines (Tables 3, 4 and 5, respectively). This approach allowed us to identify differentially expressed genes with a common trend in expression among the cell lines compared.

**Table 3:** Genes differentially expressed in common among colon cancer cell lines resistant to MTX

GenBank ID	Gene name	Description	Ratio HT29	Ratio Caco-2
AI144299	*DHFR*	Dihydrofolate reductase	7.25	46.35
BC005238	*FXYD3*	FXYD domain containing ion transport regulator 3	7.24	2.20
BC003584	*DHFR*	Dihydrofolate reductase	6.96	50.23
BC000192	*DHFR*	Dihydrofolate reductase	6.89	38.31
NM_002380	*MATN2*	Matrilin 2	6.62	4.70
BU078629	*ZFYVE16*	Zinc finger, FYVE domain containing 16	6.06	22.73
NM_001975	*ENO2*	Enolase 2 (gamma, neuronal)	5.98	2.04
NM_017954	*CADPS2*	Ca^2+^-dependent activator protein for secretion 2	5.60	2.27
AI991103	*AXIIR*	Similar to annexin II receptor	5.14	2.03
NM_000791	*DHFR*	Dihydrofolate reductase	4.71	21.17
U05598	*AKR1C2*	Aldo-keto reductase family 1, member C2	4.63	10.15
M33376	*AKR1C2*	Aldo-keto reductase family 1, member C2	4.41	8.89
NM_012242	*DKK1*	Dickkopf homolog 1	4.25	2.56
NM_014867	*KBTBD11*	Kelch repeat and BTB (POZ) domain containing 11	4.22	2.14
AB037848	*KIAA1427*	Synaptotagmin XIII	4.13	9.56
NM_014733	*ZFYVE16*	Zinc finger, FYVE domain containing 16	4.11	15.6
NM_001353	*AKR1C1*	Aldo-keto reductase family 1, member C1	3.94	8.87
NM_002439	*MSH3*	MutS homolog 3	3.87	4.00
S68290	*AKR1C1*	Aldo-keto reductase family 1, member C1	3.66	9.45
J04810	*MSH3*	MutS homolog 3	3.27	8.23
NM_000691	*ALDH3A1*	Aldehyde dehydrogenase 3 family, member A1	2.86	3.81
AI718385	*SLC26A2*	Solute carrier family 26 member 2	2.76	2.02
NM_003069	*SMARCA1*	SWI/SNF related, regulator of chromatin a1	2.60	2.49
AB029026	*TACC1*	Transforming, acidic coiled-coil containing protein 1	2.35	3.89
NM_006283	*TACC1*	Transforming, acidic coiled-coil containing protein 1	2.31	3.26
AF188298	*DAB2*	Disabled homolog 2	2.29	2.11
NM_020299	*AKR1B10*	Aldo-keto reductase family 1, member B10	2.26	22.95
BC006471	*MLLT11*	Myeloid/lymphoid or mixed-lineage leukemia	2.18	2.60
W93554	*SH3PXD2A*	SH3 and PX domains 2A	2.14	3.45
NM_014778	*NUPL1*	Nucleoporin like 1	0.46	0.43
NM_006033	*LIPG*	Lipase, endothelial	0.40	0.42
NM_012338	*TM4SF12*	Transmembrane 4 superfamily member 12	0.39	0.38
NM_007150	*ZNF185*	Zinc finger protein 185 (LIM domain)	0.38	0.42
AB014605	*MAGI2*	Membrane associated guanylate kinase	0.38	0.30
AB033831	*SCDGF*	Platelet derived growth factor C	0.35	0.35
NM_021021	*SNTB1*	Syntrophin, beta 1	0.35	0.23
NM_021822	*APOBEC3G*	Apolipoprotein B, catalytic polypeptide-like 3G	0.30	0.08
AB039791	*ARP11*	Actin-related protein Arp11	0.29	0.46
NM_013352	*SART2*	Squamous cell carcinoma antigen	0.22	0.31
NM_004362	*CLGN*	Calmegin	0.21	0.19
AI912583	*GLIPR1*	GLI pathogenesis-related 1 (glioma)	0.21	0.45
Z19574	*KRT17*	Keratin 17	0.19	0.46
NM_003186	*TAGLN1*	Transgelin	0.19	0.14
NM_014059	*RGC32*	Response gene to complement 32	0.17	0.44
BE872674	*CLEC3A*	C-type lectin domain family 3, member A	0.15	0.24
NM_003212	*TDGF1/3*	Teratocarcinoma-derived growth factor 1/3	0.13	0.47
BC000069	*RARRES2*	Retinoic acid receptor responder 2	0.07	0.33
AF110400	*FGF19*	Fibroblast growth factor 19	0.06	0.26
NM_006851	*GLIPR1*	GLI pathogenesis-related 1	0.05	0.28
NM_006169	*NNMT*	Nicotinamide N-methyltransferase	0.04	0.24
AF208043	*IFI16*	Interferon, gamma-inducible protein 16	0.03	0.38
BG256677	*IFI16*	Interferon, gamma-inducible protein 16	0.02	0.40
NM_006169	*NNMT*	Nicotinamide N-methyltransferase	0.02	0.24

Genes differentially expressed by at least twofold with a *P*-value < 0.05 including multiple testing correction were compared between HT29 and Caco-2 cells using Venn diagrams in GeneSpring GX software v 7.3.1. The table includes the GenBank IDs for all genes, their respective common names and the associated description. The ratio column corresponds to the fold change in expression of each gene relative to its sensitive counterpart.

**Table 4:** Genes differentially expressed in common among breast cancer cell lines resistant to MTX

GenBank	Gene name	Description	Ratio MCF7	Ratio MDA-MB-468
NM_019093	*UGT1A1/3/4/5/6/7/8/9/10*	UDP glucuronosyltransferase 1, polypeptides A1/3/4/5/6/7/8/9/10	24.36	27.93
NM_000463	*UGT1A1/4/6/8/9/10*	UDP glucuronosyltransferase 1, polypeptides A1/4/6/8/9/10	15.31	17.66
NM_021027	*UGT1A1/4/6/8/9/10*	UDP glucuronosyltransferase 1, polypeptides A1/4/6/8/9/10	13.55	17.05
NM_001072	*UGT1A1/3/4/5/6/7/8/9/10*	UDP glucuronosyltransferase 1, polypeptides A1/3/4/5/6/7/8/9/10	13.21	16.88
AV691323	*UGT1A1/3/4/5/6/7/8/9/10*	UDP glucuronosyltransferase 1, polypeptides A1/3/4/5/6/7/8/9/10	13.05	16.82

Genes differentially expressed by at least twofold with a *P*-value < 0.05 including multiple testing correction were compared between MCF7 and MDA-MB-468 cells as described in Table 3.

**Table 5:** Genes differentially expressed in common among colon cancer cell lines resistant to MTX

GenBank	Gene name	Description	Ratio MIA PaCa-2	Ratio K562	Ratio Saos-2
BC003584	*DHFR*	Dihydrofolate reductase	16.97	17.78	4.06
BC000192	*DHFR*	Dihydrofolate reductase	12.56	13.57	5.28
AI144299	*DHFR*	Dihydrofolate reductase	12.1	9.45	8.94
NM_000791	*DHFR*	Dihydrofolate reductase	9.66	6.76	5.57
BE622627	*PIK3R3*	Phosphoinositide-3-kinase, regulatory subunit 3	5.23	2.09	4.44
AW469790	*EEF1A1*	Eukaryotic translation elongation factor 1 alpha 1	2.29	2.75	2.05
NM_012446	*SSBP2*	Single-stranded DNA binding protein 2	2.2	5.04	2.03
AF318326	*APG10L*	ATG10 autophagy related 10 homolog	2.08	7.39	2.19

Genes differentially expressed by at least twofold with a *P*-value < 0.05 including multiple testing correction were compared between MIA PaCa-2, K562 and Saos-2 cells as described in Table 3.

### Detection of nodes upon generation of biological association networks

**Figure 2:**
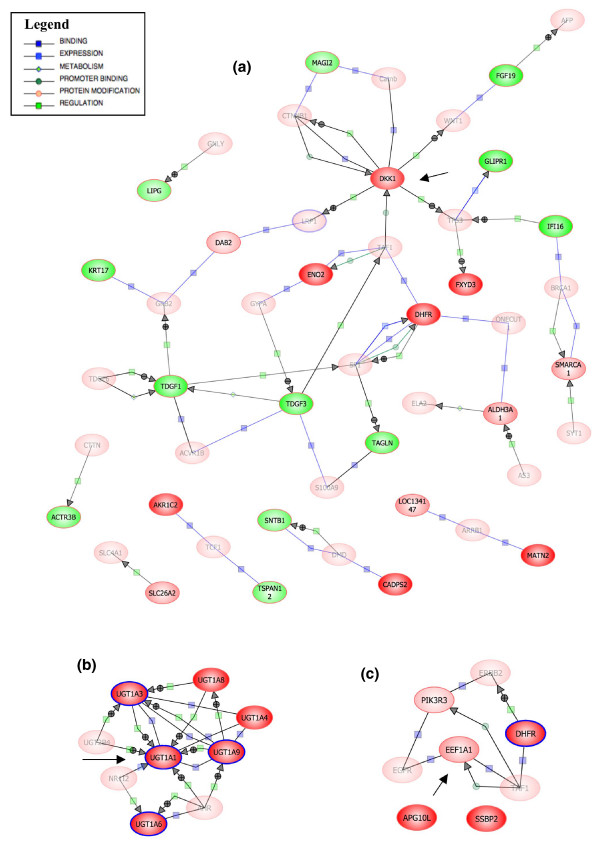
BANs of differentially expressed genes in common between cell lines. The lists of common genes between both colon cancer cell lines, between both breast cancer cell lines, and among the other three cell lines studied (representative of pancreatic cancer, leukemia and osteosarcoma) were used to construct BANs with the Pathway Architect software. Expanded networks were constructed for each list - **(a) **colon cancer, **(b) **breast cancer and **(c) **the other three cell lines - by setting an advanced filter that included the categories of binding, expression, metabolism, promoter binding, protein modification and regulation (see legend). Only proteins are represented. Overlapping of the expression levels was also performed (red for overexpressed genes and green for underexpressed genes; translucent shading represents genes that were not in the list and were added by the program from the interactions database). The BANs show some node genes that were studied further (those with arrows pointing to them).

**Figure 3:**
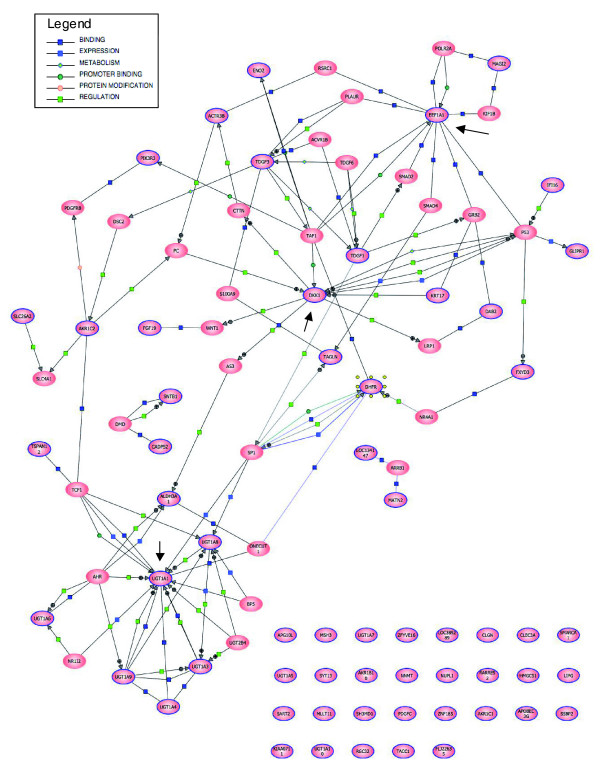
BAN of all common genes. A BAN was constructed as previously described with all the genes included in any of the three lists of common genes (encircled in blue). Genes added by the program from the interaction database are not outlined. Node genes are those with arrows pointing to them.

BANs were constructed using the Pathway Architect software as described in Methods starting with the lists of genes differentially expressed in common between both colon cancer cell lines, both breast cancer cell lines and among the pancreas cancer, leukemia and osteosarcoma cell lines (Figure 2a,b,c, respectively). In the BANs generated, *DKK1 *is a highly interconnected node in the colon cancer cell lines, *UGT1A *family members formed a network of genes differentially expressed in breast cancer, and *EEF1A1 *was commonly overexpressed in pancreatic cancer, leukemia and osteosarcoma. A BAN including all the genes of the three lists of differentially expressed genes was constructed (Figure 3). *DKK1*, *UGT1A *and *EEF1A1 *all seemed to be important nodes of this newly constructed network, and thus were selected for further study.

### Effect on MTX sensitivity of siRNAs designed against the mRNAs of node genes

**Figure 4:**
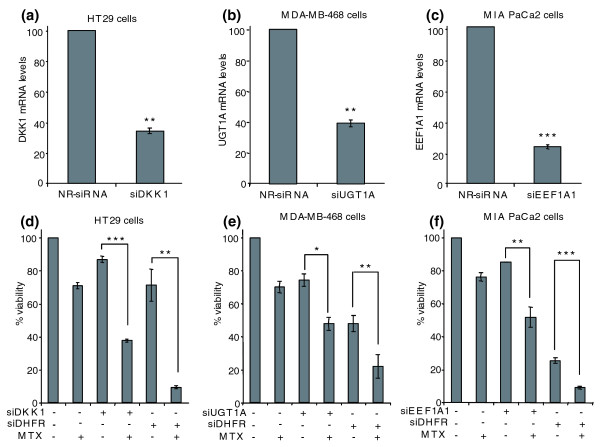
Effects on MTX sensitivity of treatment with siRNAs against *DKK1*, *UGT1A*s or *EEF1A1*. **(a-f) **Treatment with siDKK1 was performed in HT29 cells (a, d), siUGT1A was transfected in MDA-MB-468 cells (b, e) and the effects of siEEF1A1 were determined in MIA PaCa-2 cells (c, f). Treatments were performed as described in Methods, and MTX was added after 48 h. Cell viability was determined 3 days after MTX treatment (d-f). (d-f) A siRNA against *dhfr *was transfected in each of the three cell lines, and its effects on cell viability are presented. For determination of mRNA levels, real-time RT-PCR was performed with 500 ng of total RNA extracted 48 h after siRNA transfection (a-c). All results are expressed as percentages relative to the non-related negative control siRNA (NR-siRNA). Values are the mean of three independent experiments ± SE. *P < 0.05, **P < 0.01 and ***P < 0.001.

Given that the node genes *DKK1*, *UGT1A*s and *EEF1A1 *were overexpressed in cells resistant to MTX (Table 6), we investigated the effect of decreasing their mRNA levels by means of siRNAs on the sensitivity to this chemotherapeutic agent. We also performed treatments with siDHFR in order to assess the role of DHFR in MTX resistance. HT29 and MDA-MB-468 cell lines were used as models of colon and breast cancer, respectively, and MIA PaCa-2 cells were selected as the model for the other three cell lines. Previously, it was confirmed that the mRNA levels of the three genes were decreased 48 hours after siRNA treatment (Figure 4a-c). Cells were pre-incubated with individual siRNAs for 48 hours before the addition of methotrexate. The presence of 100 nM of either siDKK1 or siDHFR caused increases in MTX cytotoxicity in HT29 cells of 50% and 65%, respectively, compared to MTX alone (Figure 4d). Incubation with 100 nM of either siUGT1A or siDHFR in MDA-MB-468 cells caused increases in cytotoxicity of 36% and 50%, respectively, compared to MTX alone (Figure 4e). Treatment of MIA PaCa-2 cells with 30 nM of either siEEF1A1 or siDHFR resulted in increased cytotoxicity of 35% and 65%, respectively, compared to MTX alone (Figure 4f). The same approach was conducted in the resistant counterparts of the HT29, MDA-MB-468 and MIA PaCa-2 cell lines, but no significant changes in cytotoxicity were observed (*P *> 0.05; data not shown).

**Table 6:** Validation of *DKK1*, *UGT1A *and *EEF1A1 *overexpression in the resistant cells

		Expression	
		
Gene	Cell line	Microarray	RT-PCR validation
*DKK1*	HT29	4.25	5.66 ± 0.23
	Caco-2	2.56	1.96 ± 0.03
*UGT1A*	MCF-7	15.90	23.41 ± 0.94
	MDA-MB-468	19.27	16.28 ± 0.19
*EEF1A1*	MIA PaCa-2	2.29	3.89 ± 0.16
	K562	2.75	2.38 ± 0.47
	Saos-2	2.05	1.85 ± 0.15

The expression levels for the three node genes (*DKK1*, *UGT1A *and *EEF1A1*) derived from both the microarray data (average of the overexpressed *UGT1A *family members) and real-time RT-PCR are given. All the results are expressed as fold changes relative to the sensitive cells and values are the mean of triplicate experiments ± SE.

Given that the UGT1A family is involved in the metabolism of other drugs, we also performed combination treatments with siUGT1A and SN38, the active metabolite of the anticancer drug irinotecan. Transfection of the siRNA was performed as described above, and 1 nM SN38 was added 48 hours after siRNA treatment. These incubations led to a significant (*P *< 0.01) increase of 46% in SN38 sensitivity.

A non-related siRNA against the luciferase gene was used as a negative control in all experiments. Transfection of this siRNA was performed in parallel with the other siRNAs, and was used to normalize the results.

### DKK1 is overexpressed in HT29 MTX-resistant cells due to higher activation of the Wnt pathway

**Figure 5:**
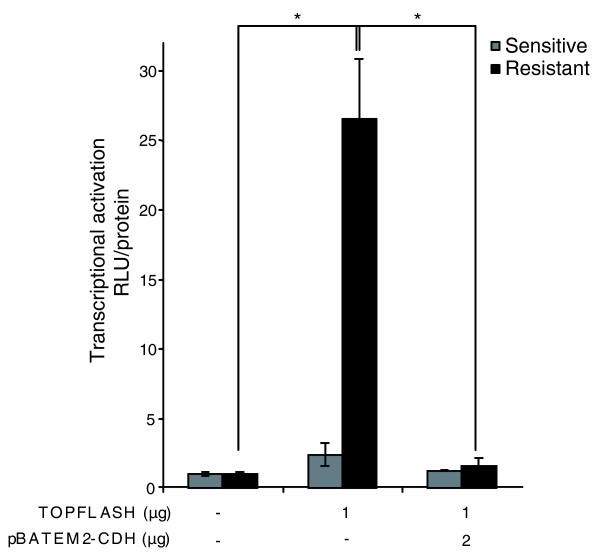
Transcriptional activation of the Wnt pathway in MTX-resistant cells leads to *DKK1 *overexpression. HT29 cells, either sensitive or resistant, were transiently transfected with 1 μg of a luciferase reporter of β-catenin-mediated transcriptional activation (TOPFLASH) using Fugene™ HD in the presence or absence of 2 μg of E-cadherin expression vector (pBATEM2-CDH). Thirty hours after transfection, luciferase activity (relative light units (RLU)) was assayed. The protein content was used to normalize the luciferase activity for each sample and is expressed relative to that of pGL3 basic vector (mean ± standard error of the mean for triplicate wells). *P < 0.05.

As *DKK1 *is known to be transcriptionally regulated by the Wnt pathway, we investigated the degree of activation of this signaling pathway in MTX-resistant HT29 cells compared with their sensitive counterpart. Cells were transiently transfected with the reporter plasmid TOPFLASH, bearing three T-cell factor (TCF) binding sites. A transcriptional activation of 26-fold resulted from the transfection of TOPFLASH in the resistant cells, while no significant activation was observed upon transfection in the sensitive cells (Figure 5). Additionally, co-transfections of TOPFLASH with an expression plasmid for E-cadherin (pBATEM2-CDH) were performed in both cell lines. As shown in Figure 5, overexpression of E-cadherin in the resistant cells led to a marked decrease in TOPFLASH activity, down to basal activity. No significant changes in transcriptional activity were observed when these co-transfections were performed in the sensitive cells.

## Discussion

The main objective of this work was to explore whether node genes could be identified from BANs constructed starting from genes differentially expressed in MTX-resistant cells from different human cancer cell lines, representative of five tissues. Those putative node genes may then be used as targets to increase the sensitivity toward MTX.

We started by determining and comparing the patterns of differential gene expression associated with MTX resistance in seven cell lines. The only differentially expressed gene in common among all the cell lines studied was *dhfr*. Its overexpression, at both the mRNA and protein levels, was confirmed in the MTX-resistant cells studied. The mRNA upregulation can be explained either by gene amplification of the *dhfr *locus, a well recognized mechanism for MTX resistance [15-17], or by an increase of *dhfr *transcription rate. In accordance with this, HT29, CaCo-2, MCF-7 and MIA PACA-2 resistant cells display an increased *dhfr *copy number. Indeed, amplification of the 5q14 locus, including *dhfr *and flanking genes, has been described in HT29 MTX-resistant cells [13]. On the other hand, the other cell lines studied bear no changes in *dhfr *copy number. Thus, drug resistance in MDA-MB-468, K562 and Saos-2 cells may be caused by any of the other known mechanisms for MTX resistance [18-22]. Additionally, one has to take into account that MTX causes the differential expression of many genes that may be direct or indirect regulators of cell proliferation, survival or apoptosis, and that this expression pattern can contribute to modulation of the resistance phenotype. As described in this work, the overexpression of *DKK1*, *UGT1A*s or *EEF1A1 *could represent a mechanism, parallel to *DHFR *overexpression, that plays a role in MTX resistance; the possible contribution of the overexpression of each of these genes is addressed below. Other work has determined genes that correlate with the capability of parental cells to resist treatment using concentrations assessed to be clinically achievable in tumor tissue [23]. Although this approach is different to the goal of our work, to identify genes differentially expressed in cells with acquired resistance to high concentrations of MTX, we compared the results obtained by Gyorffy *et al*. [23] with ours and found only six genes in common, namely *CD99*, *CKMT1*, *DHRS2*, *IGFBP7*, *MAP7 *and *MYO1E*.

Hierarchical clustering of all samples indicated that the MTX-resistant cells and their sensitive counterparts were highly correlated with regards to gene expression with each other. This is in accordance with reports showing that two breast tumor samples from the same patient before and after treatment with doxorubicin pair together in a hierarchical clustering [24]. Moreover, the same authors proposed that the molecular program of a primary tumor could generally be retained in its metastases. Similar results were obtained with leukemic cells from patients [25].

Interestingly, as shown in Figure 1, the gene expression patterns for the cell lines from the same tissue origin were very similar. Sets of coordinately expressed genes provide gene expression signatures that can indicate where to find targets suitable for gene therapy. Thus, we generated a list of genes differentially expressed in common between the two colon cancer cell lines. This list included, among others, genes encoding DHFR, the target for MTX, three members of the AKR family and ENO2, which we have previously studied as modulators of MTX resistance [8,13], and DKK1.

BAN construction using the genes differentially expressed in common between both colon cancer cell lines identified *DKK1 *as a highly interconnected node of the network, which could as such be a candidate druggable gene. DKK1 is a secreted protein involved in embryonic development [26] and is classically considered to function as an inhibitor of the canonical Wnt signaling pathway [27] (see [28] for a review). However, it does not take an active part in the Wnt/β-catenin pathway in colon cancer cells, as mutation of adenomatous polyposis coli (one of the components of Wnt pathway) occurs in most human colon cancers [29,30], thus disconnecting the effector part of the signaling cascade from the Wnt receptors, where DKK1 exerts its inhibitory effect [31,32]. This situation led us to hypothesize that DKK1 could have other cellular functions aside from its role in the Wnt pathway. Indeed, a role for DKK1 overexpression in cancer [26], including hepatobastomas [33] and breast cancer bone metastasis [34], aggressive tumors, epithelial-mesenchymal transition [35] and proliferation [36], has been previously suggested, although its precise mechanism of action has not yet been elucidated. In the case of HT29 MTX-resistant cells, the role of DKK1 is unclear, although it seems to be related to the resistant phenotype, since treatment with the siRNA against *DKK1 *mRNA showed a chemosensitization toward MTX. In keeping with this, Katula and collaborators [37] showed that folate deficiency led to the downregulation of DKK1, and that MTX inhibited *DKK1 *transcription. Thus, *DKK1 *overexpression in HT29 MTX-resistant cells could constitute a mechanism to overcome the transcriptional repression exerted by MTX.

It is worth noting that we had previously proposed the activation of the Wnt/β-catenin pathway to be an important step in MTX resistance in HT29 colon cancer cells [13]. In this cell line, E-cadherin is chromosomically lost and underexpressed, thus allowing β-catenin to play its function in gene transcription. In order to shed some light on the possible role of the Wnt pathway in the overexpression of *DKK1 *in HT29 resistant cells, we performed transient transfection experiments with a luciferase reporter of β-catenin-mediated transcriptional activation. These experiments showed that the Wnt pathway was more active in the HT29 resistant cells than in HT29 sensitive cells, and that re-expression of E-cadherin in the resistant cells resulted in lower β-catenin-mediated transcriptional activation, probably due to recruitment of β-catenin to the adherent junctions. Interestingly, *DKK1 *is transcriptionally regulated by Wnt/β-catenin signaling. Thus, constitutive activation of this signaling pathway through β-catenin, downstream of adenomatous polyposis coli, could represent a mechanism for *DKK1 *overexpression in HT29 MTX-resistant cells.

*UGT1A*s were the only genes differentially expressed in common between both breast cancer cell lines analyzed. UGTs comprise a family of membrane glycoproteins that come from one single gene located on chromosome 2q37, rendering nine functional UGT1A proteins by alternative splicing of 13 different first exons with the common exons 2 to 5 [38]. UGTs are involved in phase II metabolism of a wide range of metabolites, both endogenous and exogenous [39]. Glucuronidation is an important metabolic process, as it carries out the biotransformation of lipophilic substrates into hydrophilic glucuronides, which can be more easily removed from the body. Among the different products that can undergo glucuronidation, we find analgesics, sex hormones, flavonoids, rifampicin, bilirubin and tobacco-specific carcinogens [40,41]. Some anticancer drugs, such as topotecan, irinotecan, SN-38 (the active metabolite of irinotecan), doxorubicin and 4-hydroxytamoxifen, have also been described to be substrates of UGT1A activity [42-47]. Metabolism by UGT1A family members has been described to induce resistance toward daunorubicin (in both cell lines and rat hyperplastic liver nodules) [48,49], mycophenolic acid [50,51], mitoxantrone [52], SN-38 [53,54], camptothecin [55] and other drugs [56,57]. Indeed, drug inactivation by metabolism within tumor cells is recognized as an important mechanism of drug resistance, and, specifically, glucuronidation by UGT enzymes has been proposed to contribute to multidrug resistance of several chemotherapeutic drugs [56]. From our results using a siRNA against *UGT1A *mRNA we can point out a role for this family of genes in MTX resistance in MDA-MB-468 breast cancer cells. In fact, Hanioka *et al*. [58] reported that β-naphthoflavone induced *UGT1A *mRNA levels. This could be a feasible explanation for the *UGT1A *overexpression we observed in the breast cancer MTX-resistant cells used in our study.

*EEF1A1 *was overexpressed in common among MIA PaCa-2, K562 and Saos-2 resistant cell lines. EEF1A1 is a ubiquitously expressed protein elongation factor that recruits amino-acetylated tRNAs to the A site of the ribosome (see [59] for a review). Although it has been traditionally described as a cellular housekeeper enzyme, overexpression of EEF1A1 is found in melanomas and tumors of the pancreas, breast, lung, prostate and colon [59,60]. It has been demonstrated that EEF1A expression is related to increased cell proliferation [61,62], oncogenic transformation [63], delayed cell senescence [64] and metastasis [65]. Moreover, increased EEF1A1 expression has been related to cisplatin [66], doxorubicin [67] and MTX resistance [68], maybe due to its ability to inhibit apoptosis [69]. It has been proposed that EEF1A overexpression promotes cell growth and replication by contributing to an overall increase in protein translation. Antisense-mediated abrogation of *EEF1A1 *expression inhibits tumorigenesis and anchorage-independent cell replication in prostate tumor cells [70]. Our functional analyses using siRNA technology against *EEF1A1 *are in keeping with these results, and show a chemosensitization of MIA PaCa-2 cells, thus stating a role for EEF1A1 in MTX resistance in this cell line.

In summary, our results provide evidence that node genes can be identified by constructing BANs with lists of genes differentially expressed in common between cell lines resistant to MTX. RNA interference technology has enabled us to demonstrate a role for DKK1, UGT1As and EEF1A1 in MTX resistance.

## Conclusions

BANs were constructed using genes differentially expressed in common between cells resistant to MTX from seven human cancer cell lines representative of five tissues. We have been able to identify important node genes in the BANs, namely *DKK1 *in colon cancer cells, *UGT1A*s in breast cancer cells and *EEF1A1 *in pancreatic cancer, leukemia and osteosarcoma cells. These three genes were functionally validated using siRNAs against their respective mRNAs, which resulted in increased sensitivity to MTX.

## Abbreviations

APRT: Adenine Phosphoribosyltransferase; BAN: biological association network; DHFR: dihydrofolate reductase; DKK1: Dikkopf homolog 1; EEF1A1: eukaryotic translation elongation factor 1 alpha 1; MTT: 3-(4,5-Dimethylthiazol-2-yl)-2,5-diphenyltetrazolium bromide; MTX: methotrexate; NLP: Natural Language Processing; SE: standard error; siRNA: small interfering RNA; TCF: T-cell factor; UGT: UDP glucuronosyl transferase.

## Competing interests

The authors declare that they have no competing interests.

## Authors' contributions

ES participated in microarray data analyses, BAN generation and cell treatment with siRNAs. CO carried out the determination of *DHFR *mRNA levels, *dhfr *copy number and protein levels. SR performed the luciferase experiments. CA generated MDA-MB-468 cells resistant to MTX. VN helped with data interpretation and drafting the manuscript, critically revising it. CJC conceived the study, participated in microarray data analyses and in BAN generation. All authors read and approved the final manuscript.

## Additional data files

The following additional data are available with the online version of this paper: a table listing of genes differentially expressed at least twofold in HT29 MTX-resistant cells (Additional data file 1); a table listing genes differentially expressed at least twofold in Caco-2 MTX-resistant cells (Additional data file 2); a table listing genes differentially expressed at least twofold in MCF-7 MTX-resistant cells (Additional data file 3); a table listing genes differentially expressed at least twofold in MDA-MD-468 MTX-resistant cells (Additional data file 4); a table listing genes differentially expressed at least twofold in MIA PaCa-2 MTX-resistant cells (Additional data file 5); a table listing genes differentially expressed at least twofold in K562 MTX-resistant cells (Additional data file 6); a table listing genes differentially expressed at least twofold in Saos-2 MTX-resistant cells (Additional data file 7).

## Supplementary Material

Additional data file 1 The list was generated using GeneSpring software v 7.3.1. It includes the GenBank numbers of all genes, their respective common names and the associated description. The fold change values relative to the control (sensitive cells) are provided. The differentially expressed transcripts corresponding to open reading frames, transcribed sequences, cDNA clones or hypothetical genes have been omitted. Out of the 54,675 transcripts contained in the microarray, 21,216 passed the control strength filter, 3,046 the Benjamini and Hochberg false discovery rate-corrected *P *< 0.05 filter, and 518 remained after filtering for at least a twofold expression difference (the genes presented in this file). Click here for file

Additional data file 2 The list was generated using GeneSpring software v 7.3.1. Out of the 54,675 transcripts contained in the microarray, 21,304 passed the control strength filter, 5,399 the Benjamini and Hochberg false discovery rate-corrected *P *< 0.05 filter, and 848 remained after filtering for at least a twofold expression difference (the genes presented in this file). Click here for file

Additional data file 3 The list was generated using GeneSpring software v 7.3.1. Out of the 54,675 transcripts contained in the microarray, 21,438 passed the control strength filter, 3,052 the Benjamini and Hochberg false discovery rate-corrected *P *< 0.05 filter, and 1,002 remained after filtering for at least a twofold expression difference (the genes presented in this file). Click here for file

Additional data file 4 The list was generated using GeneSpring software v 7.3.1. Out of the 54,675 transcripts contained in the microarray, 27,498 passed the control strength filter, 12 the Benjamini and Hochberg false discovery rate-corrected *P *< 0.05 filter, and all of them remained after filtering for at least a twofold expression difference (the genes presented in this file). Click here for file

Additional data file 5 The list was generated using GeneSpring software v 7.3.1. Out of the 54,675 transcripts contained in the microarray, 21,340 passed the control strength filter, 7,612 the Benjamini and Hochberg false discovery rate-corrected *P *< 0.05 filter, and 2,248 remained after filtering for at least a twofold expression difference (the genes presented in this file). Click here for file

Additional data file 6 The list was generated using GeneSpring software v 7.3.1. Out of the 54,675 transcripts contained in the microarray, 24,964 passed the control strength filter, 5,805 the Benjamini and Hochberg false discovery rate-corrected *P *< 0.05 filter, and 490 remained after filtering for at least a twofold expression difference (the genes presented in this file). Click here for file

Additional data file 7 The list was generated using GeneSpring software v 7.3.1. Out of the 54,675 transcripts contained in the microarray, 20,520 passed the control strength filter, 7,592 the Benjamini and Hochberg false discovery rate-corrected *P *< 0.05 filter, and 2,383 remained after filtering for at least a twofold expression difference (the genes presented in this file). Click here for file

## References

[B1] Huttenhower C Flamholz AI Landis JN Sahi S Myers CL Olszewski KL Hibbs MA Siemers NO Troyanskaya OG Coller HA Nearest Neighbor Networks: clustering expression data based on gene neighborhoods. BMC Bioinformatics 2007 8 250 10.1186/1471-2105-8-250 17626636 PMC1941745

[B2] Yu JX Sieuwerts AM Zhang Y Martens JW Smid M Klijn JG Wang Y Foekens JA Pathway analysis of gene signatures predicting metastasis of node-negative primary breast cancer. BMC Cancer 2007 7 182 10.1186/1471-2407-7-182 17894856 PMC2077336

[B3] Kyoto Encyclopedia of Genes and Genomes http://www.genome.jp/kegg/

[B4] Dohr S Klingenhoff A Maier H Hrabe de Angelis M Werner T Schneider R Linking disease-associated genes to regulatory networks via promoter organization. Nucleic Acids Res 2005 33 864 872 10.1093/nar/gki230 15701758 PMC549397

[B5] Brazhnik P de la Fuente A Mendes P Gene networks: how to put the function in genomics. Trends Biotechnol 2002 20 467 472 10.1016/S0167-7799(02)02053-X 12413821

[B6] Natarajan J Berrar D Dubitzky W Hack C Zhang Y DeSesa C Van Brocklyn JR Bremer EG Text mining of full-text journal articles combined with gene expression analysis reveals a relationship between sphingosine-1-phosphate and invasiveness of a glioblastoma cell line. BMC Bioinformatics 2006 7 373 10.1186/1471-2105-7-373 16901352 PMC1557675

[B7] Melera PW Acquired versus intrinsic resistance to methotrexate: diversity of the drug-resistant phenotype in mammalian cells. Semin Cancer Biol 1991 2 245 255 1912529

[B8] Selga E Noe V Ciudad CJ Transcriptional regulation of aldo-keto reductase 1C1 in HT29 human colon cancer cells resistant to methotrexate: role in the cell cycle and apoptosis. Biochem Pharmacol 2008 75 414 426 10.1016/j.bcp.2007.08.034 17945194

[B9] Rocke DM Durbin B A model for measurement error for gene expression arrays. J Comput Biol 2001 8 557 569 10.1089/106652701753307485 11747612

[B10] Bader GD Donaldson I Wolting C Ouellette BF Pawson T Hogue CW BIND - The Biomolecular Interaction Network Database. Nucleic Acids Res 2001 29 242 245 10.1093/nar/29.1.242 11125103 PMC29820

[B11] Zanzoni A Montecchi-Palazzi L Quondam M Ausiello G Helmer-Citterich M Cesareni G MINT: a Molecular INTeraction database. FEBS Lett 2002 513 135 140 10.1016/S0014-5793(01)03293-8 11911893

[B12] Mosmann T Rapid colorimetric assay for cellular growth and survival: application to proliferation and cytotoxicity assays. J Immunol Methods 1983 65 55 63 10.1016/0022-1759(83)90303-4 6606682

[B13] Selga E Morales C Noe V Peinado MA Ciudad CJ Role of Caveolin 1, E-Cadherin, Enolase 2 and PKCalpha on resistance to methotrexate in human HT29 colon cancer cells. BMC Med Genomics 2008 1 35 10.1186/1755-8794-1-35 18694510 PMC2527490

[B14] Gene Expression Omnibus http://www.ncbi.nlm.nih.gov/geo/

[B15] Carman MD Schornagel JH Rivest RS Srimatkandada S Portlock CS Duffy T Bertino JR Resistance to methotrexate due to gene amplification in a patient with acute leukemia. J Clin Oncol 1984 2 16 20 6583326 10.1200/JCO.1984.2.1.16

[B16] Curt GA Cowan KH Chabner BA Gene amplification in drug resistance: of mice and men. J Clin Oncol 1984 2 62 64 6699659 10.1200/JCO.1984.2.1.62

[B17] Alt FW Kellems RE Schimke RT Synthesis and degradation of folate reductase in sensitive and methotrexate-resistant lines of S-180 cells. J Biol Chem 1976 251 3063 3074 944697

[B18] Yu M Melera PW Allelic variation in the dihydrofolate reductase gene at amino acid position 95 contributes to antifolate resistance in Chinese hamster cells. Cancer Res 1993 53 6031 6035 8261418

[B19] Srimatkandada S Schweitzer BI Moroson BA Dube S Bertino JR Amplification of a polymorphic dihydrofolate reductase gene expressing an enzyme with decreased binding to methotrexate in a human colon carcinoma cell line, HCT-8R4, resistant to this drug. J Biol Chem 1989 264 3524 3528 2914962

[B20] Jansen G Mauritz R Drori S Sprecher H Kathmann I Bunni M Priest DG Noordhuis P Schornagel JH Pinedo HM Peters GJ Assaraf YG A structurally altered human reduced folate carrier with increased folic acid transport mediates a novel mechanism of antifolate resistance. J Biol Chem 1998 273 30189 30198 10.1074/jbc.273.46.30189 9804775

[B21] Roy K Tolner B Chiao JH Sirotnak FM A single amino acid difference within the folate transporter encoded by the murine RFC-1 gene selectively alters its interaction with folate analogues. Implications for intrinsic antifolate resistance and directional orientation of the transporter within the plasma membrane of tumor cells. J Biol Chem 1998 273 2526 2531 10.1074/jbc.273.5.2526 9446553

[B22] Zhao R Sharina IG Goldman ID Pattern of mutations that results in loss of reduced folate carrier function under antifolate selective pressure augmented by chemical mutagenesis. Mol Pharmacol 1999 56 68 76 10385685

[B23] Gyorffy B Surowiak P Kiesslich O Denkert C Schafer R Dietel M Lage H Gene expression profiling of 30 cancer cell lines predicts resistance towards 11 anticancer drugs at clinically achieved concentrations. Int J Cancer 2006 118 1699 1712 10.1002/ijc.21570 16217747

[B24] Perou CM Sorlie T Eisen MB Rijn M van de Jeffrey SS Rees CA Pollack JR Ross DT Johnsen H Akslen LA Fluge O Pergamenschikov A Williams C Zhu SX Lonning PE Borresen-Dale AL Brown PO Botstein D Molecular portraits of human breast tumours. Nature 2000 406 747 752 10.1038/35021093 10963602

[B25] Alizadeh AA Eisen MB Davis RE Ma C Lossos IS Rosenwald A Boldrick JC Sabet H Tran T Yu X Powell JI Yang L Marti GE Moore T Hudson J Jr Lu L Lewis DB Tibshirani R Sherlock G Chan WC Greiner TC Weisenburger DD Armitage JO Warnke R Levy R Wilson W Grever MR Byrd JC Botstein D Brown PO Distinct types of diffuse large B-cell lymphoma identified by gene expression profiling. Nature 2000 403 503 511 10.1038/35000501 10676951

[B26] Forget MA Turcotte S Beauseigle D Godin-Ethier J Pelletier S Martin J Tanguay S Lapointe R The Wnt pathway regulator DKK1 is preferentially expressed in hormone-resistant breast tumours and in some common cancer types. Br J Cancer 2007 96 646 653 10.1038/sj.bjc.6603579 17245340 PMC2360041

[B27] Rothbacher U Lemaire P Creme de la Kremen of Wnt signalling inhibition. Nat Cell Biol 2002 4 E172 173 10.1038/ncb0702-e172 12105428

[B28] Nelson WJ Nusse R Convergence of Wnt, beta-catenin, and cadherin pathways. Science 2004 303 1483 1487 10.1126/science.1094291 15001769 PMC3372896

[B29] Polakis P Wnt signaling and cancer. Genes Dev 2000 14 1837 1851 10921899

[B30] Gonzalez-Sancho JM Aguilera O Garcia JM Pendas-Franco N Pena C Cal S Garcia de Herreros A Bonilla F Munoz A The Wnt antagonist DICKKOPF-1 gene is a downstream target of beta-catenin/TCF and is downregulated in human colon cancer. Oncogene 2005 24 1098 1103 10.1038/sj.onc.1208303 15592505

[B31] Semenov MV Tamai K Brott BK Kuhl M Sokol S He X Head inducer Dickkopf-1 is a ligand for Wnt coreceptor LRP6. Curr Biol 2001 11 951 961 10.1016/S0960-9822(01)00290-1 11448771

[B32] Mao B Wu W Davidson G Marhold J Li M Mechler BM Delius H Hoppe D Stannek P Walter C Glinka A Niehrs C Kremen proteins are Dickkopf receptors that regulate Wnt/beta-catenin signalling. Nature 2002 417 664 667 10.1038/nature756 12050670

[B33] Koch A Waha A Hartmann W Hrychyk A Schuller U Wharton KA Jr Fuchs SY von Schweinitz D Pietsch T Elevated expression of Wnt antagonists is a common event in hepatoblastomas. Clin Cancer Res 2005 11 4295 4304 10.1158/1078-0432.CCR-04-1162 15958610

[B34] Voorzanger-Rousselot N Goehrig D Journe F Doriath V Body JJ Clezardin P Garnero P Increased Dickkopf-1 expression in breast cancer bone metastases. Br J Cancer 2007 97 964 970 17876334 10.1038/sj.bjc.6603959 PMC2360424

[B35] Monaghan AP Kioschis P Wu W Zuniga A Bock D Poustka A Delius H Niehrs C Dickkopf genes are co-ordinately expressed in mesodermal lineages. Mech Dev 1999 87 45 56 10.1016/S0925-4773(99)00138-0 10495270

[B36] Gregory CA Singh H Perry AS Prockop DJ The Wnt signaling inhibitor dickkopf-1 is required for reentry into the cell cycle of human adult stem cells from bone marrow. J Biol Chem 2003 278 28067 28078 10.1074/jbc.M300373200 12740383

[B37] Katula KS Heinloth AN Paules RS Folate deficiency in normal human fibroblasts leads to altered expression of genes primarily linked to cell signaling, the cytoskeleton and extracellular matrix. J Nutr Biochem 2007 18 541 552 10.1016/j.jnutbio.2006.11.002 17320366

[B38] Mackenzie PI Owens IS Burchell B Bock KW Bairoch A Belanger A Fournel-Gigleux S Green M Hum DW Iyanagi T Lancet D Louisot P Magdalou J Chowdhury JR Ritter JK Schachter H Tephly TR Tipton KF Nebert DW The UDP glycosyltransferase gene superfamily: recommended nomenclature update based on evolutionary divergence. Pharmacogenetics 1997 7 255 269 10.1097/00008571-199708000-00001 9295054

[B39] Guillemette C Pharmacogenomics of human UDP-glucuronosyltransferase enzymes. Pharmacogenomics J 2003 3 136 158 10.1038/sj.tpj.6500171 12815363

[B40] Strassburg CP Manns MP Tukey RH Expression of the UDP-glucuronosyltransferase 1A locus in human colon. Identification and characterization of the novel extrahepatic UGT1A8. J Biol Chem 1998 273 8719 8726 10.1074/jbc.273.15.8719 9535849

[B41] Kiang TK Ensom MH Chang TK UDP-glucuronosyltransferases and clinical drug-drug interactions. Pharmacol Ther 2005 106 97 132 10.1016/j.pharmthera.2004.10.013 15781124

[B42] Nagar S Remmel RP Uridine diphosphoglucuronosyltransferase pharmacogenetics and cancer. Oncogene 2006 25 1659 1672 10.1038/sj.onc.1209375 16550166

[B43] McCague R Parr IB Leclercq G Leung OT Jarman M Metabolism of tamoxifen by isolated rat hepatocytes. Identification of the glucuronide of 4-hydroxytamoxifen. Biochem Pharmacol 1990 39 1459 1465 10.1016/0006-2952(90)90427-M 2334445

[B44] Platzer P Schaden S Thalhammer T Hamilton G Rosenberg B Silgoner I Jager W Biotransformation of topotecan in the isolated perfused rat liver: identification of three novel metabolites. Anticancer Res 1998 18 2695 2700 9703931

[B45] Rosing H van Zomeren DM Doyle E Bult A Beijnen JH O-glucuronidation, a newly identified metabolic pathway for topotecan and N-desmethyl topotecan. Anticancer Drugs 1998 9 587 592 10.1097/00001813-199808000-00002 9773801

[B46] Andersen A Holte H Slordal L Pharmacokinetics and metabolism of doxorubicin after short-term infusions in lymphoma patients. Cancer Chemother Pharmacol 1999 44 422 426 10.1007/s002800050999 10501917

[B47] Iyer L King CD Whitington PF Green MD Roy SK Tephly TR Coffman BL Ratain MJ Genetic predisposition to the metabolism of irinotecan (CPT-11). Role of uridine diphosphate glucuronosyltransferase isoform 1A1 in the glucuronidation of its active metabolite (SN-38) in human liver microsomes. J Clin Invest 1998 101 847 854 10.1172/JCI915 9466980 PMC508633

[B48] Cowan KH Batist G Tulpule A Sinha BK Myers CE Similar biochemical changes associated with multidrug resistance in human breast cancer cells and carcinogen-induced resistance to xenobiotics in rats. Proc Natl Acad Sci USA 1986 83 9328 9332 10.1073/pnas.83.24.9328 3540935 PMC387131

[B49] Gessner T Vaughan LA Beehler BC Bartels CJ Baker RM Elevated pentose cycle and glucuronyltransferase in daunorubicin-resistant P388 cells. Cancer Res 1990 50 3921 3927 2112982

[B50] Franklin TJ Jacobs V Jones G Ple P Bruneau P Glucuronidation associated with intrinsic resistance to mycophenolic acid in human colorectal carcinoma cells. Cancer Res 1996 56 984 987 8640790

[B51] Franklin TJ Jacobs VN Jones G Ple P Human colorectal carcinoma cells in vitro as a means to assess the metabolism of analogs of mycophenolic acid. Drug Metab Dispos 1997 25 367 370 9172956

[B52] Rekha GK Sladek NE Multienzyme-mediated stable and transient multidrug resistance and collateral sensitivity induced by xenobiotics. Cancer Chemother Pharmacol 1997 40 215 224 10.1007/s002800050649 9219504

[B53] Takahashi T Fujiwara Y Yamakido M Katoh O Watanabe H Mackenzie PI The role of glucuronidation in 7-ethyl-10-hydroxycamptothecin resistance in vitro. Jpn J Cancer Res 1997 88 1211 1217 9473740 10.1111/j.1349-7006.1997.tb00351.x PMC5921346

[B54] Cummings J Ethell BT Jardine L Boyd G Macpherson JS Burchell B Smyth JF Jodrell DI Glucuronidation as a mechanism of intrinsic drug resistance in human colon cancer: reversal of resistance by food additives. Cancer Res 2003 63 8443 8450 14679008

[B55] Brangi M Litman T Ciotti M Nishiyama K Kohlhagen G Takimoto C Robey R Pommier Y Fojo T Bates SE Camptothecin resistance: role of the ATP-binding cassette (ABC), mitoxantrone-resistance half-transporter (MXR), and potential for glucuronidation in MXR-expressing cells. Cancer Res 1999 59 5938 5946 10606239

[B56] Meijerman I Beijnen JH Schellens JH Combined action and regulation of phase II enzymes and multidrug resistance proteins in multidrug resistance in cancer. Cancer Treat Rev 2008 34 505 520 10.1016/j.ctrv.2008.03.002 18413281

[B57] Zembutsu H Ohnishi Y Tsunoda T Furukawa Y Katagiri T Ueyama Y Tamaoki N Nomura T Kitahara O Yanagawa R Hirata K Nakamura Y Genome-wide cDNA microarray screening to correlate gene expression profiles with sensitivity of 85 human cancer xenografts to anticancer drugs. Cancer Res 2002 62 518 527 11809704

[B58] Hanioka N Obika N Nishimura M Jinno H Tanaka-Kagawa T Saito K Kiryu K Naito S Narimatsu S Inducibility of UDP-glucuronosyltransferase 1As by beta-naphthoflavone in HepG2 cells. Food Chem Toxicol 2006 44 1251 1260 10.1016/j.fct.2006.01.019 16545899

[B59] Thornton S Anand N Purcell D Lee J Not just for housekeeping: protein initiation and elongation factors in cell growth and tumorigenesis. J Mol Med 2003 81 536 548 10.1007/s00109-003-0461-8 12898041

[B60] Alon U Barkai N Notterman DA Gish K Ybarra S Mack D Levine AJ Broad patterns of gene expression revealed by clustering analysis of tumor and normal colon tissues probed by oligonucleotide arrays. Proc Natl Acad Sci USA 1999 96 6745 6750 10.1073/pnas.96.12.6745 10359783 PMC21986

[B61] Hassell JA Engelhardt DL The regulation of protein synthesis in animal cells by serum factors. Biochemistry 1976 15 1375 1381 10.1021/bi00652a004 1259943

[B62] Grassi G Scaggiante B Farra R Dapas B Agostini F Baiz D Rosso N Tiribelli C The expression levels of the translational factors eEF1A 1/2 correlate with cell growth but not apoptosis in hepatocellular carcinoma cell lines with different differentiation grade. Biochimie 2007 89 1544 1552 10.1016/j.biochi.2007.07.007 17825975

[B63] Tatsuka M Mitsui H Wada M Nagata A Nojima H Okayama H Elongation factor-1 alpha gene determines susceptibility to transformation. Nature 1992 359 333 336 10.1038/359333a0 1383827

[B64] Shepherd JC Walldorf U Hug P Gehring WJ Fruit flies with additional expression of the elongation factor EF-1 alpha live longer. Proc Natl Acad Sci USA 1989 86 7520 7521 10.1073/pnas.86.19.7520 2508089 PMC298096

[B65] Edmonds BT Wyckoff J Yeung YG Wang Y Stanley ER Jones J Segall J Condeelis J Elongation factor-1 alpha is an overexpressed actin binding protein in metastatic rat mammary adenocarcinoma. J Cell Sci 1996 109 2705 2714 8937988 10.1242/jcs.109.11.2705

[B66] Johnsson A Zeelenberg I Min Y Hilinski J Berry C Howell SB Los G Identification of genes differentially expressed in association with acquired cisplatin resistance. Br J Cancer 2000 83 1047 1054 10.1054/bjoc.2000.1420 10993653 PMC2363570

[B67] Bertram J Palfner K Hiddemann W Kneba M Overexpression of ribosomal proteins L4 and L5 and the putative alternative elongation factor PTI-1 in the doxorubicin resistant human colon cancer cell line LoVoDxR. Eur J Cancer 1998 34 731 736 10.1016/S0959-8049(97)10081-8 9713282

[B68] Beyer-Sehlmeyer G Hiddemann W Wormann B Bertram J Suppressive subtractive hybridisation reveals differential expression of serglycin, sorcin, bone marrow proteoglycan and prostate-tumour-inducing gene I (PTI-1) in drug-resistant and sensitive tumour cell lines of haematopoetic origin. Eur J Cancer 1999 35 1735 1742 10.1016/S0959-8049(99)00202-6 10674022

[B69] Talapatra S Wagner JD Thompson CB Elongation factor-1 alpha is a selective regulator of growth factor withdrawal and ER stress-induced apoptosis. Cell Death Differ 2002 9 856 861 10.1038/sj.cdd.4401078 12107828

[B70] Su Z Goldstein NI Fisher PB Antisense inhibition of the PTI-1 oncogene reverses cancer phenotypes. Proc Natl Acad Sci USA 1998 95 1764 1769 10.1073/pnas.95.4.1764 9465091 PMC19182

